# Lamina Cribrosa Defects and Optic Disc Morphology in Primary Open Angle Glaucoma with High Myopia

**DOI:** 10.1371/journal.pone.0115313

**Published:** 2014-12-22

**Authors:** Yugo Kimura, Tadamichi Akagi, Masanori Hangai, Kohei Takayama, Tomoko Hasegawa, Kenji Suda, Munemitsu Yoshikawa, Hiroshi Yamada, Hideo Nakanishi, Noriyuki Unoki, Hanako Ohashi Ikeda, Nagahisa Yoshimura

**Affiliations:** Department of Ophthalmology and Visual Sciences, Kyoto University Graduate School of Medicine, 54 Kawahara-cho, Shogoin, Sakyo-ku, Kyoto, 606-8507, Japan; Cardiff University, United Kingdom

## Abstract

**Purpose:**

To investigate whether lamina cribrosa (LC) defects are associated with optic disc morphology in primary open angle glaucoma (POAG) eyes with high myopia.

**Methods:**

A total of 129 POAG patients and 55 age-matched control subjects with high myopia were evaluated. Three-dimensional scan images obtained by swept source optical coherence tomography were used to detect LC defects. Radial B-scans and infrared images obtained by spectral domain optical coherence tomography were used to measure β-peripapillary atrophy (PPA) lengths with and without Bruch's membrane (BM) (temporal, nasal, superior, and inferior), tilt angle (vertical and horizontal), and disc diameter (transverse and longitudinal). Peripapillary intrachoroidal cavitations (PICCs), disc area, ovality index, and cyclotorsion of the optic disc were analyzed as well.

**Results:**

LC defects were found in 70 of 129 (54.2%) POAG eyes and 1 of 55 (1.8%) control eyes (P<0.001). Age, sex, spherical equivalent, axial length, intraocular pressure, and central corneal thickness were not significantly different among POAG eyes with LC defects, POAG eyes without LC defects, and control eyes. Temporal PPA lengths without BM in all three groups correlated significantly with vertical and horizontal tilt angles, although no PPA length with BM correlated significantly with any tilt angle. PICCs were detected more frequently in POAG eyes with LC defects than those without LC defects (P = 0.01) and control eyes (P = 0.02). POAG eyes with LC defects showed a smaller ovality index (P = 0.004), longer temporal PPA without BM (P<0.001), and larger vertical/horizontal tilt angles (vertical, P<0.001; horizontal, P = 0.01), and transverse diameter (P = 0.01). In multivariate analysis for the presence of LC defects, presence of POAG (P<0.001) and vertical tilt angle (P<0.001) were identified as significant.

**Conclusions:**

The presence of LC defects was associated with myopic optic disc morphology in POAG eyes with high myopia.

## Introduction

Myopia is one of the most common ocular abnormalities across the globe, and its prevalence is increasing among young people, especially in East Asian countries [1]–[3]. One meta-analysis that included many participants identified myopia as a risk factor in open angle glaucoma [4], with risk increasing in association with the degree of myopia [5]. In primary open angle glaucoma (POAG), visual field (VF) defects are usually detected peripherally during early stages of the disease, while the paracentral area of the visual field is spared until the end stages. However, the clinical manifestation of POAG with high myopia differs from that of POAG with typical myopia. POAG with high myopia is associated with more retinal nerve fiber layer (RNFL) defects within the papillomacular bundle, which can lead to the development of a paracentral scotoma during the early stages of disease [6], [7]. In advanced stages, VF defects are typically located temporally and inferior to the fixation point [8], posing a substantial threat to the patient's quality of vision.

In a myopic patients with POAG, the optic disc show various morphological changes such as large and tilted disc, with cyclotorsion of the longer axis and extensive peripapillary atrophy (PPA) [9]–[11]. PPA was reported to be associated with the presence [12], progression [13], [14], and location of VF defects [15] in POAG eyes. Recent SD-OCT research has shown that conventionally defined β-PPA can be distinguished by the presence or absence of Bruch's membrane. Additionally, PPA without Bruch's membrane is associated with axial globe elongation and myopic optic disc morphologic changes [16], [17]. Moreover, optic disc deformation and direct scleral compression or stretching at the peripapillary region in degenerative myopia could lead to progressive VF defects that overlap with those characteristic of POAG [18], [19].

The lamina cribrosa (LC), which is a principal site of axonal damage in glaucoma, is a multilayered sieve-like supporting structure within the optic disc [20], [21]. Structural changes to the LC, such as thinning [22], [23], posterior displacement [24], and the creation of large pores [25], have been reported as characteristic of POAG eyes. Previous reports have shown that the LC appears as a hyperreflective structure on enhanced depth images (EDI) obtained using spectral domain optical coherence tomography (SD-OCT) and swept source OCT (SS-OCT). It has also been reported that this continuous LC hyperreflectivity has focal defects called, “focal LC defects”, in subset of eyes with POAG [26], [27], [28], [29], [30]. Focal LC defects were more likely to be found in eyes with NTG [28], a past history of disc hemorrhage [28], [29], inferior mean deviation (MD) values [28], and negative refractive error [28]. Furthermore, the locations of these focal defects corresponded strongly with those of RNFL defects in the same eye [30]. However, the mechanism of focal LC defect formation has not yet been elucidated.

SS-OCT exhibits less signal decay over a given depth as compared with SD-OCT, which makes the former approach advantageous for imaging deep structures. SS-OCT is reportedly just as effective as EDI-SD-OCT for visualizing LC in myopic glaucomatous eyes [31], [32]. Here we examine optic discs in eyes with high myopia using SS-OCT and SD-OCT, and investigate whether focal LC defects are associated with optic disc morphology in myopic eyes.

## Methods

### Ethics statement

All of the procedures adhered to the tenets of the Declaration of Helsinki, and the study was approved by the Institutional Review Board and Ethics Committee of Kyoto University Graduate School of Medicine. The nature of the study and its possible consequences were explained to study candidates, after which written informed consent was obtained from all who participated.

### Participants

The medical records of patients who were observed at the Glaucoma clinic at the Department of Ophthalmology, Kyoto University from October 2011 through December 2013 were reviewed. All participants had undergone complete ophthalmic examinations including the measurement of best-corrected visual acuity (BCVA) with a 5 M Landolt chart; refraction; slit-lamp biomicroscopy; intraocular pressure (IOP) measurements with a Goldman applanation tonometer; gonioscopy; central corneal thickness (CCT) measurements with an ultrasonic pachymeter (SP-3000, Tomay, Tokyo, Japan); axial length measurements by partial laser interferometry (IOLMaster, Carl Zeiss Meditec, Dublin, CA); dilated biomicroscopic examination; stereoscopic color optic disc photography (3-Dx simultaneous stereo disc camera, Nidek, Gamagori, Japan); imaging using a Heidelberg Retina Tomography 2 (HRT 2, Heidelberg Engineering), a prototype SS-OCT system (Topcon, Tokyo, Japan), SD-OCT (Spectralis-OCT, Heidelberg Engineering, Dossenheim, Germany) and standard automated perimetry (SAP) using the Swedish interactive threshold algorithm standard 24-2 protocol for the Humphrey Visual Field Analyzer (Carl Zeiss Meditec, Dublin, CA). The value for refractive error was converted to the spherical equivalent. The average IOP for the last three examinations was used for analysis.

The inclusion criteria were: BCVA of ≥20/30 (Snellen equivalent), spherical equivalent <−6.0 diopters, normal anterior segment, normal and open angle by gonioscopy, glaucomatous appearance of the optic disc, glaucomatous visual field defects corresponding to a glaucomatous optic disc appearance and reliable HFA results with a false-positive error rate <15%, a false-negative error rate <15%, and fixation loss <20%. Eyes meeting ≥2 of the following criteria were considered to have glaucomatous VF defects: (1) a cluster of 3 points with probability <5% on a pattern deviation map in 1 or more hemifields, including 1 point or more with probability <1% or 2 points with probability <1%; (2) glaucoma hemifield test results outside normal limits; and (3) pattern standard deviation <5%.The exclusion criteria were hazy media, evidence of vitreoretinal disease including diabetic retinopathy, pathologic myopic fundus changes such as patchy chorioretinal atrophy, lacquer crack lesions, or choroidal neovascularization, and previous ocular surgery. Patients with a systemic disease or a neurological disease that might cause VF defects or RNFL damage (e.g., uncontrolled hypertension, uncontrolled diabetes mellitus, pituitary tumor, and cerebral infarction) were also excluded. When both eyes of a patient were eligible for a POAG diagnosis, 1 eye was randomly selected.

Age-matched control subjects with high myopia were defined as those with spherical equivalent <−6.0 diopters, IOP <22 mmHg, a non-glaucomatous optic disc appearance and no VF defects. Normal eyes also had no history of ocular symptoms, disease, or intraocular surgery.

A paracentral scotoma was defined as a glaucomatous VF defect in one hemifield within 10° of fixation with at least 1 point with probability <1% lying at the 2 innermost paracentral points, regardless of the presence of VF defects outside the central 10° [7].

### HRT 2 optic disc area measurements

HRT 2 images were obtained from undilated pupils. Optic disc area was measured using the built-in HRT 2 software (Heidelberg Engineering, Heidelberg, Germany) as previously reported [7], [13]. The measurement was included in subsequent analyses only if the standard deviation of the mean topographic image was <40 µm. An experienced examiner, masked to the other findings, manually outlined the optic disc margin while viewing fundus color photographs. Magnification errors were corrected by the HRT2 software using the patient's refractive error and corneal curvature measurements.

### Measurements of ovality index and disc cyclotorsion

To estimate the tilt and cyclotorsion of the optic disc, we used two indices as formally reported [7], [33], [34]. An examiner studied color fundus photographs of the optic disc taken at a 45° viewing angle using custom-built software [7]. The software automatically drew an ellipse along the contour of the optic disc and a reference line from the disc center to the fovea as well as a vertical meridian from the reference line. Ovality index was defined as the ratio of minimum to maximum optic disc diameter, and cyclotorsion of the optic disc as degrees between the longer axis of the optic disc and the vertical meridian.

### SD-OCT measurements of β-PPA length, tilt angle, and optic disc diameter

β-PPA lengths were measured as previously described with modifications [17], [35]. Tomographic images of the optic disc were obtained by EDI-SD-OCT, with infrared (IR) fundus images acquired simultaneously using a confocal scanning laser ophthalmoscope (CSLO). The center of each radial B-scan image was carefully placed at the disc center. Images were obtained every 30° with scan length of 6 mm. Fifty OCT frames were averaged to yield each section of the B-scan.

β-PPA length and optic tilt angle were analyzed with the intrinsic viewer (Heidelberg Eye Explorer software version 1.7.0.0; Heidelberg Engineering). This viewer automatically synchronizes the vertical lines of each B-scan and IR image. The distance between two arbitrary points as measured with intrinsic calipers was used to correct for the effect of corneal curvature.

The examiner (YK) defined three points of measurement: the disc margin, the start of the retinal pigment epithelium (RPE), and the start of BM using IR and B-scan images magnified to 200%. The disc margin was defined as the border between low and high reflectivity in IR images. The start of the RPE and BM were identified on B-scans as the termination of continuous lines of high reflectivity. Vertical lines were drawn from these three points in the B-scan image, and the distances among the three lines were measured using calipers. PPA length with BM was defined as the distance from the RPE border to BM, and PPA length without BM was defined as the distance from BM to the disc margin. When RPE beginning or BM opening was not clearly visible due to the shadow of large vessels or thick nerve fiber layer, such case was excluded from this analysis.

**Figure 1:**
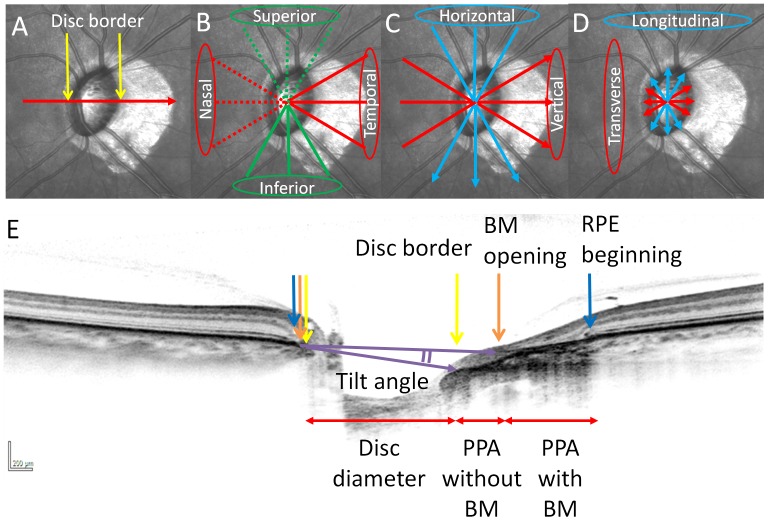
Measurement of PPA lengths, tilt angles and disc diameters with spectral-domain optical coherence tomography (SD-OCT) in the left eye. (*A–D*) Infrared (IR) images of the optic disc. (*E*) SD-OCT B-scan image oriented according to the red arrow in (*A*). Disc border (yellow arrows), RPE (blue arrows) and Bruch's membrane (BM) (orange arrows) were shown in (*A, E*). PPA lengths were categorized into superior, inferior, temporal, and nasal lenghs (*B*). Tilt angle was determined by the angle between the two lines drawn between the BM termination point and disc border (two purple arrows). Tilt angle was categorized into horizontal and vertical tilt angles (*C*). Disc diameter was defined as longitudinal and transverse diameters (*D*).

A total of 24 PPA length measurements (12 PPA lengths with and without BM, respectively) in the area surrounding the disc were classified as superior, inferior, temporal, or nasal, with three PPA length measurements averaged for each sector. For example, in the left eye, superior PPA length was calculated as the average of three radial scans obtained at 1, 11, and 12 o'clock; inferior PPA length was measured at 5, 6, and 7 o'clock; temporal PPA length was measured at 3, 4, and 5 o'clock; and nasal PPA length was measured at 8, 9, and 10 o'clock (Fig. 1A, B, E).

To measure tilt angle, a reference line based on the internal border of BM was defined in order to compensate for potential tilting of the OCT image. Using the same radial scan images, two lines were drawn between the BM and disc borders by the viewer, then captured as image data, which were imported to Image J software (National Institutes of Health, Bethesda, MD) in order to measure the angle between the lines. This angle was measured in 6 radial scan images of each eye to define tilt angle. Three radial scans sectioned from 12–6, 1–7 and 5–11 o'clock were averaged to define horizontal tilt angle. Scans extending from 2–8, 3–9 and 4–10 o'clock were averaged to determine vertical tilt angle (Fig. 1A, C, E). When the temporal disc border was posteriorly tilted with respect to the nasal disc border, the angle was considered as “positively tilted”. When the inferior disc border was tilted posteriorly with reference to the superior disc border, the angle was also considered as “positively tilted”.

To measure optic disc diameter, a line was drawn between two disc borders and the distance was measured. Three radial scans, taken at the 12 to 6, 1 to 7, and 5 to 11 o'clock positions were averaged to define longitudinal diameter. Scans extending at 2 to 8, 3 to 9, and 4 to 10 o'clock positions were averaged to determine transverse diameter (Fig. 1A, D, E).

**Figure 2:**
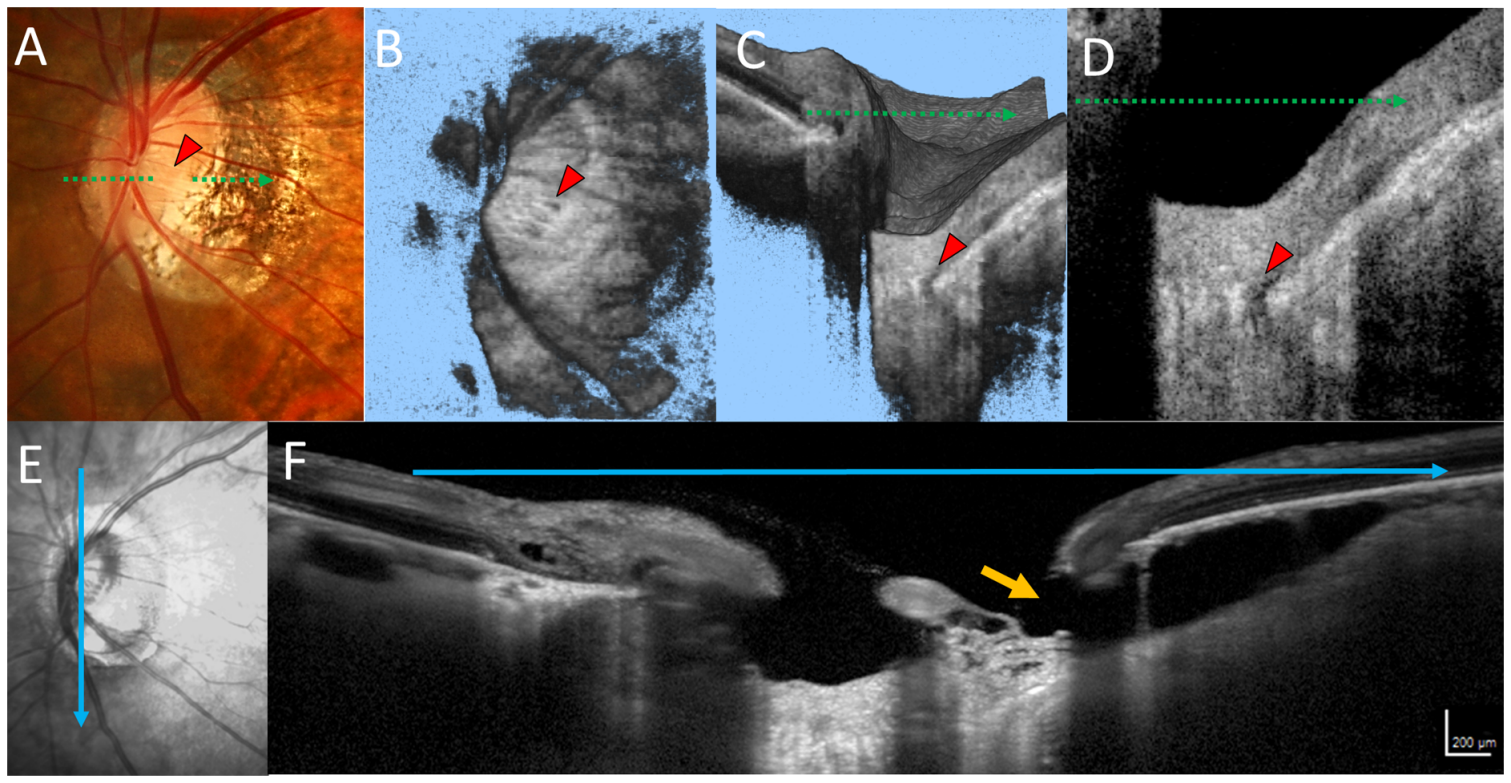
A representative case of an eye with primary open angle glaucoma, a lamina cribrosa (LC) defect, and peripapillary intrachoroidal cavities (PICCs). (*A*) A dark yellowish-orange lesion was seen in the area inferior to the optic disc. The LC defect is visible in the en face image (*B*), volume scan image (*C*), and B-scan image (*D*, red arrowhead). The infrared image (*E*) and spectral domain optical coherence tomography B-scan image (*F*), oriented at the blue arrow in (*E*), are shown. PICCs are visible as hyporeflective cavities between Bruch's membrane and the sclera. The retinal nerve fiber layer was not visible at the orange arrow.

The presence of peripapillary intrachoroidal cavitations (PICCs) was evaluated using 6 radial scan images for each eye included in the study. PICCs were defined as yellowish-orange lesions in the area surrounding the optic disc as detected in fundus photographs [36], with triangular thickening of the choroid (with the base at the optic disc border) and a distance between BM and sclera of ≥200 µm in at least 1 of 6 scan images (Fig. 2) [37].

### 3D SS-OCT image acquisition of optic disc and assessment of LC defects

Optic disc images were obtained as 3D scans using a prototype SS-OCT system, which is basically equivalent to the commercially available DRI OCT-1 (Topcon, Tokyo, Japan) [19], [29]. A 3D radial scan was acquired over a 3×3-mm area centered on the optic disc center by well-trained examiners. En face images were used in combination with vertical and horizontal serial B-scan images to evaluate laminar structure.

**Figure 3:**
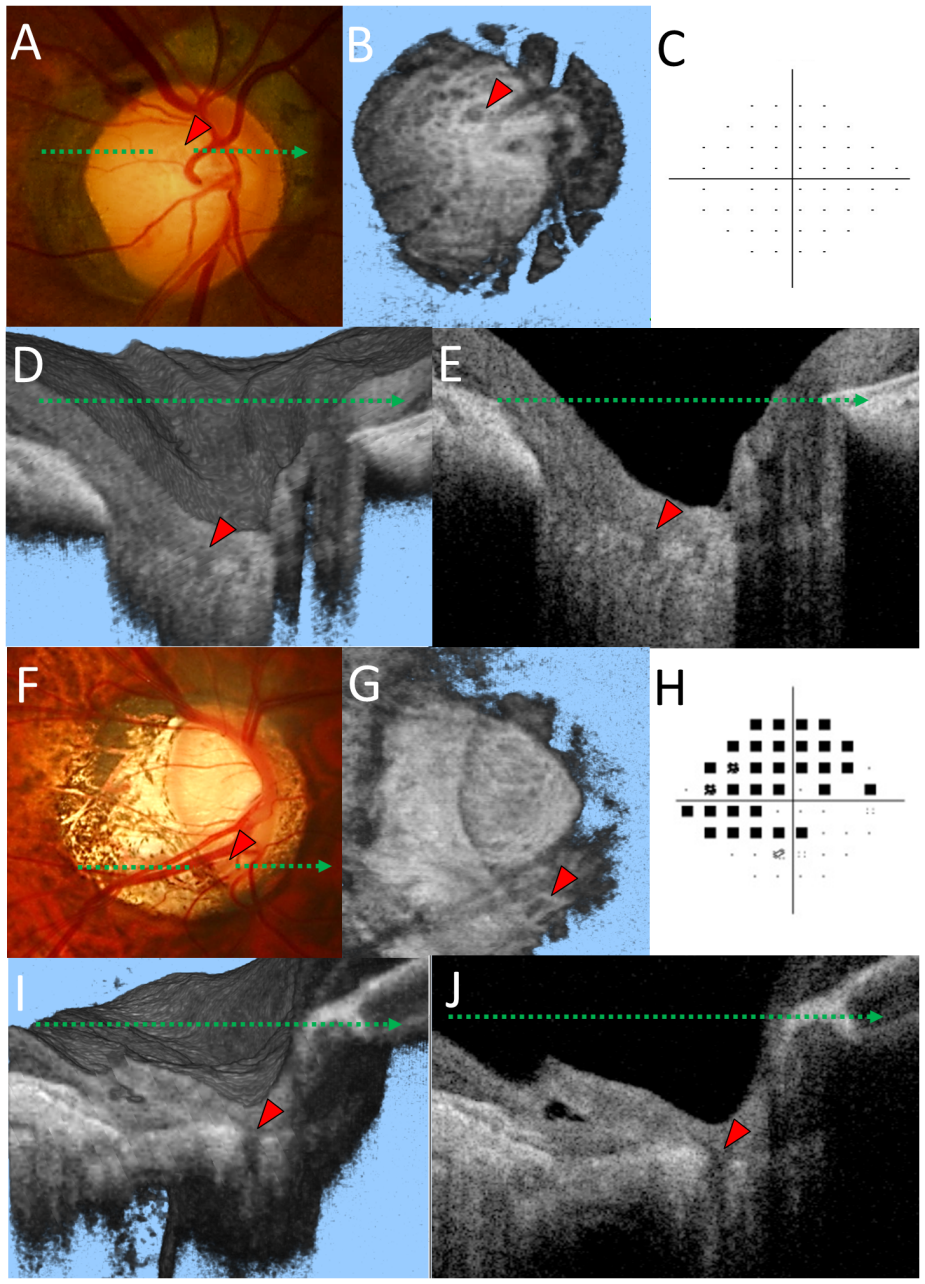
Representative cases of a healthy highly myopic eye with enlarged lamina pores (*A–E*) and an eye with primary open angle glaucoma and lamina cribrosa (LC) defects (F–J). (*A*) A lamina pore can be seen within the optic disc cup (red arrowhead) in the optic disc photograph. (*B*) Multiple hyporeflective dots and a large hyporeflective region (red arrowhead) can be interpreted as lamina pores in en face optic disc images at the level of the LC. (*C*) Humphrey 24-2 pattern deviation plots show no glaucomatous visual field defect. Volume image (*D*) and horizontal B-scan image (*E*) corresponding to the green dotted line in (*A*). Note that the lamina structure was not fully defective in the hyporeflective region. (*F*) The LC defect can be seen in the inferior border of the optic disc rim (red arrowhead). (*G*) A wedge shaped hyporeflective region (red arrowhead) is also apparent in the en face image. (*H*) Humphrey 24-2 pattern deviation plots showed a glaucomatous visual field defect. Volume image (*I*) and horizontal B scan image (*J*) corresponding to the green dotted arrow in (*F*). Note that the lamina structure was fully defective in the hyporeflective region.

The evaluation of LC defects was performed as previously described [29]. In brief, the SS-OCT image set was independently reviewed for focal LC defects by two graders (KT and YK), who were masked to all other information. An LC defect was defined as a loss of high reflectivity from the anterior-to-posterior border of the full-thickness LC on vertical/horizontal serial B-scan images and a minimum width of hyporeflectivity of >100 µm on en-face images (Figs. 2 and 3)[26].

Hyporeflective lesions in the LC suspected to be vascular shadows were excluded. If the two graders did not agree on the existence of an LC defect, they reviewed and discussed the data until a consensus was reached. If no consensus could be reached, the candidate LC defect was excluded from analysis.

Next, the location of an LC defect was defined. We selected the one en face image in which the lamina structure was most clearly visible from all images constructed by the 3D dataset. Two diagonal lines were drawn in the 3×3 mm square of the en face image. This divided the lamina structure into superior, inferior, temporal, and nasal quadrants. The LC defect location was determined by assessing in which quadrant the LC defect was located.

### Interobserver reproducibility

To evaluate the interobserver reproducibility of PPA length and tilt angle measurements, two observers (YK, KM) localized the RPE and BM borders and disc margin, then measured the lengths and angles in 100 randomly selected eyes. Intraclass correlation coefficients (ICCs) were calculated with their confidence intervals.

### Statistical analysis

A one-way analysis of variance (ANOVA) and Tukey's post-hoc test were used to compare differences in continuous variables among the groups. The frequency of PICC detection was compared using a chi-square test with Bonferroni's correction. Pearson's correlation coefficients were used to evaluate the associations among various parameters of optic disc morphology as measured using the SD-OCT, HRT2, and fundus photographs. Univariate and multivariate logistic regression analyses were performed to determine the association between each optic disc parameter and the presence of LC defects. Optic disc parameters, including disc area, ovality index, optic disc cyclotorsion, PPA length with/without BM, horizontal/vertical tilt angle, and transverse/longitudinal diameters, were the independent variables. The presence/absence of LC defects was used as the dependent variable. All statistical analyses were performed using SPSS software version 19.0 (SPSS, Chicago, IL). P values less than 0.05 were accepted as statistically significant, except for those calculated to compare PICCs.

## Results

This study initially involved 225 subjects with high myopia. Among these, 41 eyes were excluded because of poor SS-OCT or SD-OCT scan quality (n = 34), an unclear optic disc margin (n = 5), or unreliable visual field test results (n = 2). Finally, the remaining 129 eyes of 129 POAG patients with high myopia and 55 eyes of 55 age-matched control subjects were evaluated. LC defects were found in 70 of 129 POAG eyes (54.2%, Fig. 3) and 1 of 55 age-matched control eyes (1.8%). The frequencies of LC defects were significantly different between the POAG and control eyes (P<0.001).

In agreement with our previous report [29], LC defects were found as the laminar disinsertion (laminar insertion into the scleral wall, 70 of 75 [93.3%] eyes) and the laminar hole (middle portion of the laminar structure, 5 of 75 [6.7%]). In all 75 eyes, LC defects were found in the temporal portion of the LC. Moreover, LC defects were detected in the superior portion of 3 eyes and in the inferior portion of 6 eyes. Interestingly, no LC defects were found in the nasal quadrant. The location of LC defects spatially corresponded to areas of PPA in all eyes examined.

Table 1 shows the demographic information for POAG eyes with LC defects, POAG eyes without LC defects, and age-matched control subjects. MD was significantly worse in POAG eyes with or without LC defects as compared to control subjects (P<0.001 in both). MD was not significantly different between POAG eyes with LC defects and without LC defects (P = 0.09). Paracentral scotomas were detected more frequently in POAG eyes with LC defects than in POAG eyes without LC defects (P = 0.008). Age, sex, spherical equivalent, axial length, IOP, and CCT were not significantly different among the three groups. Next, the frequency of paracentral scotomas was compared in the subgroup of POAG eyes with better than MD>−6.0 dB (26 POAG eyes with LC defects and 26 POAG eyes without LC defects). Paracentral scotomas were also detected more frequently in POAG eyes with LC defects (n = 15) than in POAG eyes without LC defects (n = 6) in the subgroup (P = 0.01). MD value was not significantly different between these groups (POAG eyes with LC defects; −4.1±1.4 dB, POAG eyes without LC defects; −3.6±1.3 dB, P = 0.25).

**Table 1:** Demographics of Patients and Age-matched Control Subjects with High Myopia.

	POAG, n = 129		Age-matched Control, n = 55	P value
	with LC Defects, n = 75	without LC Defects, n = 54		
Age, years	49.0±13.9 (22 to 78)	50.2±12.7 (24 to 74)	49.4±8.9 (22 to 74)	0.88*
Male/Female, n	30/40	31/28	21/34	0.23†
Spherical Equivalent, D	−8.3±3.6 (−6.1 to −16.9)	−8.5±2.5 (−6.1 to −15.3)	−8.4±3.1 (−6.1 to −14.6)	0.89*
Axial Length, mm	27.2±1.3 (24.7 to 30.4)	27.2±1.0 (25.7 to 29.9)	27.3±1.0 (24.9 to 29.3)	0.98*
IOP, mmHg	16.4±2.2 (11.0 to 19.6)	17.2±3.1 (10.3 to 18.6)	15.0±1.6 (13.0 to 18.3)	0.25*
CCT, µm	538.5±33.4 (477 to 628)	531.9±40.4 (431 to 604)	524.9±28.6 (442 to 598)	0.16*
MD, dB	−11.2±7.4 (−1.9 to 31.4)	−8.6±7.5 (−1.4 to −30.3)	−1.4±1.7 (2.5 to 4.9)	<0.001*
with/without Paracentral Scotoma, n	52/23	25/29	NA	0.008†

Values are shown in mean ± SD (range). IOP; intra ocular pressure, CCT; central corneal thickness, MD; mean deviation, LC; lamina cribrosa.

*Comparison performed using 1-way analysis of variance with post hoc Tukey's test to compare the differences among the 3 groups.

† Comparisons were performed using the chi square test.

MD in POAG eyes with LC defects and without LC defects were significantly worse than the age-matced healthy eyes (P<0.001 in both).

The interobserver reproducibilities of PPA length and tilt angle measurements are presented in Table 2. ICCs ranged from 0.902 to 0.965, which suggested that measurements of SD-OCT scan images were reproducible between examiners.

**Table 2:** Reproducibility of SD-OCT Measurements of PPA Length and Tilt Angle (n = 184).

	ICC
PPA length with BM	
Superior	0.929
Inferior	0.917
Temporal	0.916
Nasal	0.931
PPA length without BM	
Superior	0.905
Inferior	0.911
Temporal	0.902
Nasal	0.926
Tilt angle	
Vertical	0.913
Horizontal	0.965

PPA, peripapillary atrophy; ICC, intraclass correlation coefficients.

Temporal PPA lengths without BM correlated significantly with vertical tilt angle and horizontal tilt angle in all three groups. Inferior PPA lengths without BM correlated significantly with horizontal tilt angle in all three groups. Transverse diameter correlated significantly with vertical tilt angle in all three groups. Any PPA lengths with BM did not correlate significantly with any tilt angle (Table 3).

**Table 3:** Correlation of the Disc Morphological Parameters measured with SD-OCT (POAG eyes with LC Defects/POAG eyes with LC Defects/Age-matched Control Subjects).

		Vertical Tilt Angle	Horizontal Tilt Angle
PPA Length without BM			
	Superior	0.40/0.11/0.06	−0.03/0.29/0.15
	Inferior	0.17/0.08/0.08	**0.47/0.65/0.73**
	Temporal	**0.67/0.73/0.65**	**0.37/0.40/0.31**
	Nasal	0.11/−0.12/−0.11	0.01/0.19/0.23
PPA length with BM			
	Superior	0.11/0.17/0.04	0.08/0.09/0.03
	Inferior	0.12/0.09/−0.03	−0.01/0.24/0.22
	Temporal	−0.21/0.20/0.10	0.02/0.22/0.08
	Nasal	−0.02/0.17/−0.01	0.01/0.16/0.21
Transverse Diameter		−**0.48/**−**0.47/**−**0.52**	−0.18/0.08/0.26
Longitudinal Diameter		−0.19/−0.17/−**0.47**	−0.21/−0.07/−0.09

Values are expressed correlation coefficient. Significant values (P<0.05) are shown in bold.

PPA; Peripapillary Atrophy, BM; Bruch's Membrane.

Horizontal tilt angle was significantly correlated with ovality index in all three groups but not with disc area. Vertical tilt angle significantly correlated with both ovality index and disc area in all three groups. Additionally, both transverse and longitudinal diameters were significantly correlated with ovality index and disc area in all three groups (S1 Table). The means for each morphological parameters were compared among the three groups (Table 4). Ovality index, temporal PPA length without BM, horizontal/vertical tilt angles, and transverse diameter differed significantly between POAG eyes with LC defects and the other two groups (ovality index, P = 0.004; temporal PPA length with BM, P<0.001; horizontal tilt angle, P = 0.01; vertical tilt angle, P<0.001; transverse diameter, P = 0.01).

**Table 4:** Comparison of Mean Disc Morphological Parameters of among POAG eyes with LC defects, POAG eyes without LC defects, and Age-matched Control Eyes.

		POAG (n = 129)		Age-matched Control (n = 55)	P value	post hoc		
		with LC Defects (n = 75)	without LC Defects (n = 54)		ANOVA	with vs. without LC Defects	with LC Defects vs. Control	without LC Defects vs. Control
Disc Area (mm^2^)		1.95±0.78	2.38±0.70	2.12±0.85	0.01	0.008	0.45	0.21
Ovality Index		0.72±0.11	0.80±0.11	0.78±0.15	0.004	0.005	0.05	0.71
Cyclotorsion of Disc (degrees)		11.9±22.1	0.66±37.3	14.9±35.2	0.04	0.11	0.85	0.04
PPA Length without BM (µm)								
	Superior	63.5±93.0	40.6±69.4	48.7±79.3	0.28	0.26	0.57	0.87
	Temporal	560.3±345.4	361.1±323.8	392.1±257.4	<0.001	<0.001	0.008	0.87
	Inferior	217.7±241.3	160.5±199.3	193.6±182.0	0.33	0.29	0.70	0.80
	Nasal	22.4±117.4	23.6±76.6	7.1±31.1	0.54	0.99	0.62	0.59
PPA length with BM (µm)								
	Superior	87.2±103.8	67.1±80.0	67.7±77.5	0.34	0.42	0.44	1.0
	Temporal	194.5±116.5	171.7±116.5	166.0±116.3	0.32	0.5	0.34	0.97
	Inferior	113.0±83.6	96.7±109.1	70.9±106.3	0.06	0.62	0.05	0.36
	Nasal	49.8±124.5	40.8±107.4	42.4±118.4	0.90	0.9	0.93	0.99
Horizontal Tilt Angle (degrees)		3.6±3.6	1.9±2.1	2.3±3.3	0.01	0.02	0.04	0.85
Vertical Tilt Angle (degrees)		9.5±5.6	5.7±5.0	6.3±5.9	<0.001	<0.001	0.005	0.83
Transverse Diameter (µm)		1479.1±300.0	1612.1±308.4	1612.6±256.6	0.01	0.03	0.03	1.0
Longitudinal Diameter (µm)		1585.0±271.1	1646.6±249.2	1630.4±195.7	0.32	0.34	0.55	0.94

POAG; primary open angle glaucoma, LC; lamina cribrosa, PPA; peripapillary atrophy, BM; bruch membrane, PICCs; peripapillary intrachoroidal cavitations, ANOVA; analysis of varience.

Tukey's test was used for post hoc test.

PICCs were found in 25 of 129 (19.4%) POAG eyes with high myopia and 5 of 55 (9.0%) highly myopic control eyes (P = 0.08). PICCs were found in 20 POAG eyes with LC defects, 5 POAG eyes without LC defects, and 5 control eyes (P = 0.008, Fig. 2). There were significant differences between the POAG eyes with LC defects and those without LC defects (P = 0.01) as well as between the POAG eyes with LC defects and control eyes (P = 0.01). There was no difference between the POAG eyes without LC defects and the control eyes (P = 0.91). In eyes with POAG, a paracentral scotoma occurred in 9 of 25 (36.0%) eyes with PICCs and in 61 of 104 eyes (58.7%) without PICCs. Though relatively large, this difference was not statistically significant (P = 0.24).

In the univariate analysis, the presence of LC defects was significantly associated with POAG, disc area, ovality index, temporal PPA length without BM, horizontal tilt angle, vertical tilt angle, and longitudinal diameter. The significant parameters identified by multivariate analysis were only POAG (B = 4.7, P<0.001) and vertical tilt angle (B = 0.13, P<0.001) (Table 5).

**Table 5:** Univariate and Multivariate Analysis of Disc Morphological Parameters Associated with LC Defects in High Myopic Eyes.

		Univariate		Multivariate	
		B	P value	B	P value
Presence of POAG		0.52	<0.001	4.7	<0.001
Disc Area		−0.23	0.002		0.13
Ovality Index		−0.30	<0.001		0.10
Cyclotorsion of Disc		0.07	0.33		0.12
PPA Length without BM					
	Superior	0.11	0.16		0.54
	Inferior	0.09	0.23		0.43
	Temporal	0.28	<0.001		0.54
	Nasal	0.04	0.61		0.74
PPA length with BM					
	Superior	0.10	0.18		0.55
	Inferior	0.13	0.10		0.38
	Temporal	0.09	0.21		0.42
	Nasal	0.04	0.68		0.88
Horizontal Tilt Angle		0.20	0.008		0.28
Vertical Tilt Angle		0.30	<0.001	0.13	<0.001
Transverse Diameter		−0.22	0.002		0.59
Longitudinal Diameter		−0.12	0.11		0.56

LC; lamina cribrosa, PPA; peripapillary atrophy, BM; bruch membrane.

## Discussion

LC defects were found in more than 50% of the POAG eyes with high myopia. Our report previously showed that LC defects were detected in 6.6% of POAG eyes without high myopia using the same imaging modality and detection method [29]. The frequency of LC defects was much higher in POAG eyes with high myopia than in those without high myopia. Further analysis showed that LC defects were significantly associated with disc area, ovality index, temporal PPA length without BM, and horizontal and vertical tilt angles in univariate analysis, as well as with vertical tilt angle in multivariate analysis, all of which are thought to represent myopic optic disc morphological changes. Furthermore, the existence of a paracentral scotoma was associated with the presence of LC defects in the POAG eyes with high myopia.

The formation of LC defects might be associated with temporal elongation of the optic disc, because the presence of LC defects was significantly associated with vertical tilt angle in the multivariate analysis. Additionally, all LC disinsertions were located in the temporal quadrant in all eyes with LC defects. Temporal stretching due to PPA was thought to be associated with optic disc tilt [38], and furthermore, PPA without BM results from an increase in axial length [17]. Our findings that vertical tilt angle correlated significantly with temporal PPA length without BM are compatible with these previous reports. Histological analysis has shown that LC thickness in myopic eyes decreased as PPA length increased [23]. We hypothesized that a thinner LC could be more vulnerable to morphological changes. Although we did not estimate the effects of optic disc tilting on LC thickness, a morphological change imposed in a horizontal direction across the LC contributes to the formation of LC defects in highly myopic eyes.

The creation of LC defects may involve a different mechanism in highly myopic eyes than typically myopic eyes. As mentioned above, LC defects were detected in only 6.6% of the POAG eyes without high myopia in our previous study [29]. Though our results were not directly compared with other former studies [26]–[28] because of the different methodology, Tahtham et al attributed the formation of LC defects to structural weaknesses, noting that the superior and inferior portions of the LC contain thinner connective tissue and larger pores than the temporal and nasal sectors of the LC in POAG eyes [30]. Because the LC in a highly myopic eye is thinner than in an eye without myopia [23], the LC may be more vulnerable to deformation than in a non-myopic eye. Therefore, the hypothesis proposed by Tahtham et al would not be applicable to our study population. Moreover, in a study of eyes with degenerative myopia, small laminar pits equivalent to the LC defects described here were detected by SS-OCT [40]. These studies suggested that POAG eyes with LC defects might exhibit myopic characteristics more strongly than POAG eyes without LC defects. Although it can be difficult to differentiate glaucomatous from degenerative VF defects based on a clinical assessment of optic disc appearance, our study suggests that highly myopic POAG eyes with LC defects would more strongly exhibit certain characteristics associated with degenerative myopia than those without LC defects.

Disc size could be an important parameter in LC defect formation, because discs in eyes with POAG and LC defects were significantly smaller than in the other two groups. Using an initial finite element model of the POAG eye, Bellezza et al showed that IOP stress on the LC varied with optic disc size [39]. The authors showed that LC structure was more likely to be affected by IOP as disc size increased. We speculate that myopic optic disc morphological changes may contribute more strongly than IOP to the formation of LC defects in a small optic disc. Moreover, LC structure could be vulnerable to the mechanical stress because the disc size was negatively correlated with vertical tilt angle, though HRT2 imaging may underestimate disc size in eyes with large tilt angles.

Paracentral scotomas were detected more frequently in POAG eyes with LC defects than those without LC defects even in POAG eyes with MD better than −6.0 dB, which is clinically important for the management of POAG eyes. In the current study, mean MD values in POAG eyes with LC defects were worse than those in POAG eyes without LC defects in total POAG eyes and even in POAG eyes with MD better than −6.0 dB. We could not exclude the possibility that the difference of MD value affect the frequency of paracentral scotoma, because LC defects were more frequently detected in advanced POAG eyes than early POAG eyes [28]. However, it was reported that the existence of a paracentral scotoma was associated with acquired pits of the optic nerve in POAG eyes [41], a finding that supports the results presented here. Furthermore, we previously reported that a decreased optic disc ovality index was associated with RNFL defects involving the papillomacular bundle in POAG eyes with high myopia [7], which is also consistent with our findings in the current study. Tatham et al reported that the location of LC defects corresponded strongly with the location of RNFL defects [30]. Further study would elucidate the association between RNFL defects and LC defects in POAG eyes with high myopia.

In the context of pathologic myopia, PICCs are thought to be formed by tears in the border tissue located between BM and the sclera, and to be more closely related to myopic changes rather than glaucomatous disc changes [42]–[44]. In the current study, PICCs were more frequently observed in POAG eyes with LC defects as compared to POAG eyes without LC defects and control eyes. Because eyes with POAG and LC defects had significantly larger vertical tilt angles, we speculate that PICC development may occur by a similar mechanism as that related to morphological optic disc changes resulting from LC defects. PICCs represent a clinically useful finding that can be used to predict the presence of LC defects because many PICCs can be detected by standard fundus examination. Although PICCs were not specifically seen in eyes with POAG, further examination and analyses are needed to determine if comorbid PICCs and LC defects are associated with paracentral scotomas.

This study had several limitations. First, some parameters examined are affected by optic disc tilt or globe curvature. The PPA lengths were measured linearly on plane images without considering the curvature of the globe. This method might lead to underestimation of PPA length in eyes with posteriorly sloped PPA. However, temporal elongation of the eyeball would still affect LC defects, because posteriorly sloped PPA would increase actual PPA length. Disc area would be affected by optic disc tilt because flat projections, created by HRT2 imaging, were used to make these measurements. Therefore, one should keep in mind that these parameters are potentially biased. Second, because the reference line passing through the foveal center was not considered in obtaining the radial scan images, measurements of both PPA length and disc tilt angle may have been affected by ocular cyclotorsion. The reference line used may have minimized the effects of ocular cyclotorsion. However, current SD-OCT technology does not have a radial scan mode for use in combination with such a reference line. In the future, we plan to use a reference line passing through the foveal center as well as the disc center when measuring optic disc parameters. Third, we may have overlooked the presence of LC defects in the nasal region of the optic disc, because the optic disc structures there lie below a thick nerve fiber layer and are only poorly visible, especially in eyes with tilted discs. Notably, any morphological effects on the LC would be least noticeable in the nasal quadrant. However, the limitations of current OCT technology with respect to deep structural visualization of the optic disc should not be neglected.

In conclusion, LC defects were detected in more than 50% of POAG eyes with high myopia and associated with paracentral scotomas. In highly myopic eyes, these were primarily attributed to myopic optic disc morphological changes such as PICCs. LC defects were shown to be clinically important findings in highly myopic POAG eyes and to involve a mechanism that differs in some respects from that operating in non-myopic POAG eyes. However, it remains unknown whether the development of LC defects in highly myopic eyes could be prevented by reducing IOP. Further longitudinal studies would be needed to clarify this question.

## Supporting Information

S1 Data **Specific data of each group (normal and POAG) and detail data for calculation of ICCs.**
(XLSX)

S1 Table **Correlation of the Disc Morphological Parameters Measured with SD-OCT, Color Photograph and HRT2 (POAG eyes with LC Defects/POAG eyes without LC Defects/Age-matched Control Subjects).**
(DOCX)
