# Supplementary material for: Lamina Cribrosa Defects and Optic Disc Morphology in Primary Open Angle Glaucoma with High Myopia
Source: PLoS One. 2014 Dec 22;9(12):e115313. doi: 10.1371/journal.pone.0115313 (PMC4274005; doi:10.1371/journal.pone.0115313)
Supplement: S1 Table — Correlation of the Disc Morphological Parameters Measured with SD-OCT, Color Photograph and HRT2 (POAG eyes with LC Defects/POAG eyes without LC Defects/Age-matched Control Subjects). (DOCX) [file pone.0115313.s002.docx]

| Table S1. Correlation of the Disc Morphological Parameters Measured with SD-OCT, Color Photograph and HRT2 (POAG eyes with LC Defects/POAG eyes without LC Defects/Age-matched Control Subjects) | | | | |
| --- | --- | --- | --- | --- |
|  |  | Disc Area | Ovality Index | Cyclotorsion of Disc |
| Horizontal Tilt Angle | | -0.17/-0.04/0.05 | **-0.39/-0.34**/**-0.31** | **0.36 /**0.08/**0.28** |
| Vertical Tilt Angle | | **-0.39/-0.29/-0.48** | **-0.57/-0.43/-0.45** | 0.06/0.22/0.04 |
| Transverse Diameter | | **0.84/0.69/0.84** | **0.49/0.36**/**0.39** | 0.01/-0.08/0.07 |
| Longitudinal Diameter | | **0.79/0.73/0.81** | **0.38/0.33/0.42** | -0.15/-0.02/-0.19 |
| Values are expressed correlation coefficient. Significant values (P<0.05) are shown in bold. | | | | |
